# Prognostic implication of glycolysis related gene signature in non-small cell lung cancer

**DOI:** 10.7150/jca.50274

**Published:** 2021-01-01

**Authors:** Jie Yao, Rui Li, Xiao Liu, Xijia Zhou, Jianping Li, Tingting Liu, Chen Huo, Yiqing Qu

**Affiliations:** 1 Department of Pulmonary and Critical Care Medicine, Qilu Hospital, Cheeloo College of Medicine, Shandong University, Jinan 250012, China.; 2 Department of Pulmonary and Critical Care Medicine, Qilu Hospital of Shandong University, Jinan 250012, China.

**Keywords:** non-small cell lung cancer, glycolysis, TCGA, risk score, prognosis

## Abstract

Abnormal glycolysis is one of the hallmarks of cancer and plays an important role in its development. This study was devoted to identify glycolysis related genes as prognostic biomarkers for non-small cell lung cancer (NSCLC). The mRNA expression profile and clinical follow-up data were obtained using The Cancer Genome Atlas (TCGA) database. The validation set was obtained by bootstrap method of random repeated sampling. A total of 200 glycolysis-related genes were obtained from Gene Set Enrichment Analysis (GSEA) and 46 genes were significantly associated with overall survival (OS). Five genes (PKP2, LDHA, HMMR, COL5A1 and B3GNT3) were eventually identified to calculate risk score of NSCLC patients.

The univariate and multivariate Cox regression analysis indicated that the risk score was an independent prognostic factor (training set: HR=2.126, 95% CI [1.605, 2.815], *p*<0.001; validation set: HR=2.298, 95%CI [1.450, 3.640], *p*<0.001). Patients assigned to the high-risk group were associated with poor OS compared with patients in the low-risk group (training set: *P*=7.946e-06; validation set: *P*=6.368e-07). Receiver operating characteristic (ROC) curve and stratification analysis also demonstrated the potential prognostic performance. In conclusion, we constructed a novel glycolysis related risk signature which might contribute to predicting the prognosis of NSCLC.

## Introduction

Lung cancer is one of the leading causes of cancer-related death 1, especially in developed countries, where it has ascended to number one in cancer deaths 2. NSCLC accounts for approximately 80% of all lung cancer cases 3. Despite progress in treatment, the prognosis of NSCLC remains poor, due to the lack of early identification markers 4. To date, the prognosis mainly depends on histopathologic diagnosis and tumor staging 5. However, this has limitations as patients with the same degree of progression will show distinct outcomes due to individual differences 6. Some evidence has shown that the discovery and application of molecular biomarkers can provide prognostic value 7-10. Therefore, new diagnosis markers must be urgently identified for assessing prognosis of NSCLC.

Glycolysis is one of the earliest evidence of metabolic changes in tumor 11,12. Even in oxygenated circumstances, the glycolysis activity of tumor cells is also active. This metabolic characteristic is known as Warburg effect, which shows high glucose uptake, active glycolysis and large amounts of lactic acid production 13,14. Increased glycolysis satisfies the increasing proliferation of cancer cells 15. In addition, studies have shown that enhanced glucose metabolism could alter apoptosis pathway in cancer cells 16. The oncogenic regulation of glycolysis provides us with new explanation for tumor progression 17. Many studies have found that glycolysis changes take an important role in cancer progression. For example, tumor glycolysis promotes immune evasion by participating in immunosuppressive networks 18. The tumor suppressor protein p53 has a serious effect on glucose metabolic reprogramming 19. Glucose transporter GLUT1 is involved in the uptake of glucose and has significantly lower expression in highly differentiated endometrial carcinoma, than in poorly differentiated tumors 20. However, the concrete role of glycolysis in tumorigenesis of NSCLC is poorly known. Ma et al. found that NSCLC harboring EML4-ALK rearrangements displayed higher glucose metabolism 21. Li et al. reported that the anaerobic metabolism offers new targets and orientation for tumors, especially lung cancer 22. Further work is needed to demonstrate the significance of glycolysis gene expression signatures in NSCLC.

In the present study, transcriptome profiling was downloaded from TCGA database and 200 mRNAs were identified that significantly relate to glycolysis. Furthermore, a risk predictive signature including five genes was conducted to calculate the risk of NSCLC patients and further successfully validated in the validation set. Univariate and multivariate Cox regression analysis revealed the signature was an independent predictive factor for OS. The risk score also performed better than other clinical parameters in prognosis. This study might present a new thinking for assessing the prognosis of NSCLC.

## Materials and Methods

### Data collection and processing

Detailed information of NSCLC patients' containing transcriptome profiling and clinical follow-up data, were downloaded from TCGA database on April 2, 2020. Patients meeting the following criteria were excluded: 1) patient had no survival time or survival status, 2) patient had clinical information but no gene expression data. There were 935 patients included in this study. The detailed clinical characteristics are summarized in Table 1.

### Gene set enrichment analysis

GSEA analysis was used to explore whether the selected gene sets were associated with downloaded transcriptome data 23. We determined gene sets for further investigation at normalized *p* values (*p*<0.05) and normalized enrich score (|NES|≥1).

### Establishment and validation of glycolysis related gene signature

We intersected glycolysis related genes and their expression in TCGA for matching. Differentially expressed glycolysis related genes was obtained using limma R package with |log2 FC| ≥1.5 and false discovery rate (FDR) <0.05 24. Then differentially expressed genes combined with TCGA clinical data for screening prognostic genes. We identified differentially glycolysis related genes whose expression was significantly associated with OS by univariate Cox regression analysis (P<0.05). Signature genes were ultimately determined by multivariate Cox regression analysis. The prognostic signature for NSCLC patients was established. Risk score = ([Coefficient mRNA_1_] × [Expression of mRNA_1_]) + ([Coefficient mRNA_2_] × [Expression of mRNA_2_]) + ⋯ + ([Coefficient mRNA_n_] × [Expression of mRNA_n_]). Independent analyses were applied to verify the independence of the signature. Then NSCLC patients were divided into two groups based on a median risk score. The Kaplan-Meier curve was visualized using the survival R package and survminer R package. *P* < 0.05 was regarded as statistically significant. Survival ROC R package was used to compare the ROC areas. The validation set was acquired by bootstrap method 25. Furthermore, stratification analysis also verified the prognostic implication of the signature.

### Prognostic genes expression and alteration were shown by online databases

In our study, five prognostic genes involved in the signature were all upregulated in NSCLC. The gene expression was further validated in the Oncomine database and Tumor Immune Estimation Resource (TIMER) database 26,27. The alternation of the signature genes was shown by cBioportal for Cancer Genomics 28,29.

## Results

### Establishment a prognostic signature of five glycolysis related genes in NSCLC

**Figure 1:**
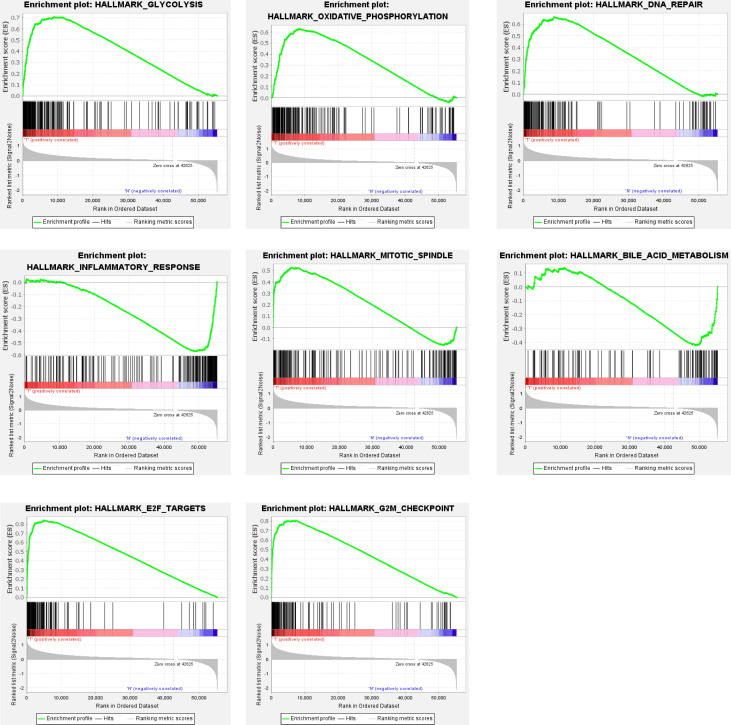
** Gene sets significantly enriched in the NSCLC using GSEA.** This includes the following: glycolysis, oxidative phosphorylation, DNA repair, inflammatory response, mitotic spindle, bile acid metabolism, E2F targets, G2M checkpoint.

**Figure 2:**
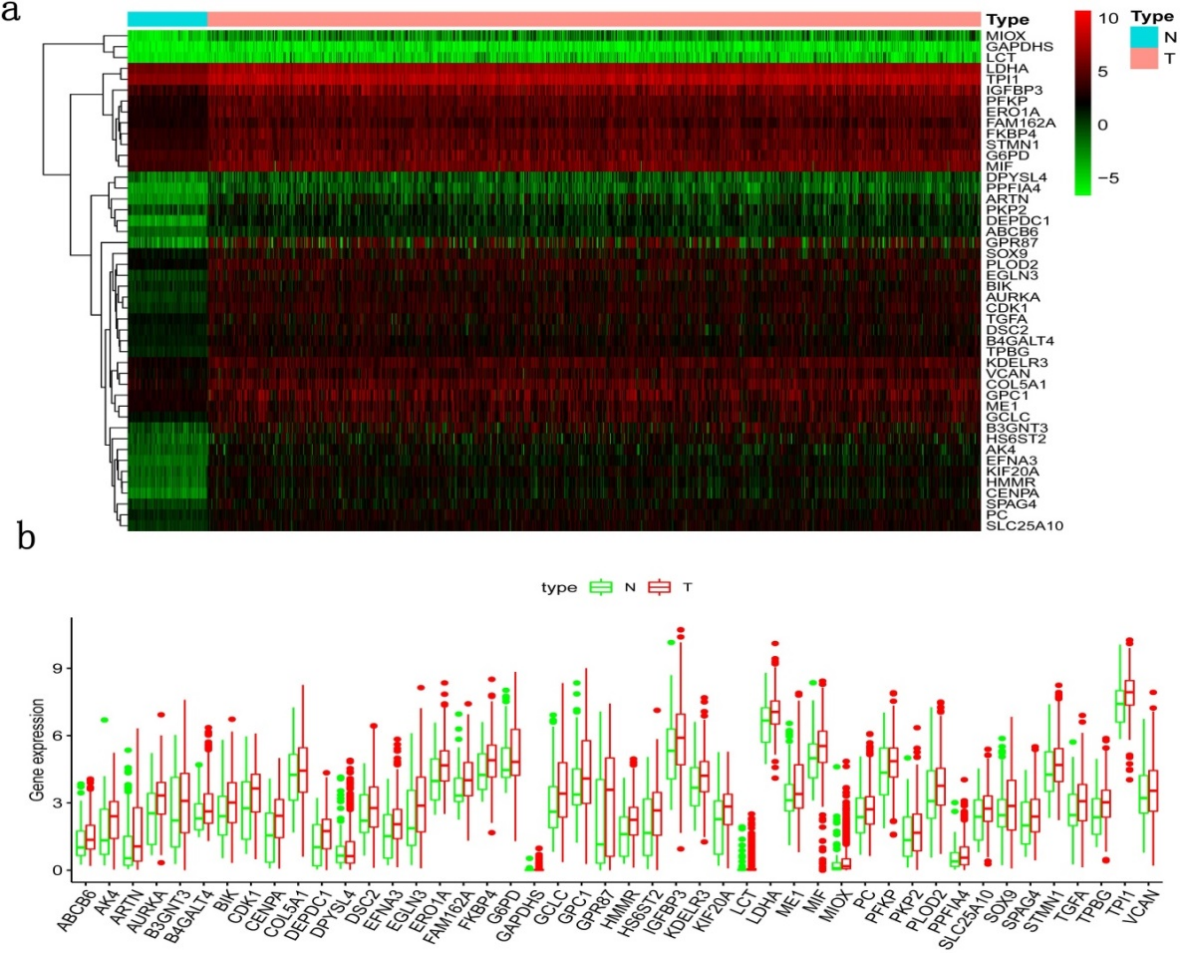
** Differentially expressed glycolysis related genes.** The red color indicates the higher gene expression value while the green color indicates the lower gene expression value. N indicates non-tumor tissues. T indicates tumor tissues**. a)** Heatmap; and,** b)** boxplot.

**Figure 3:**
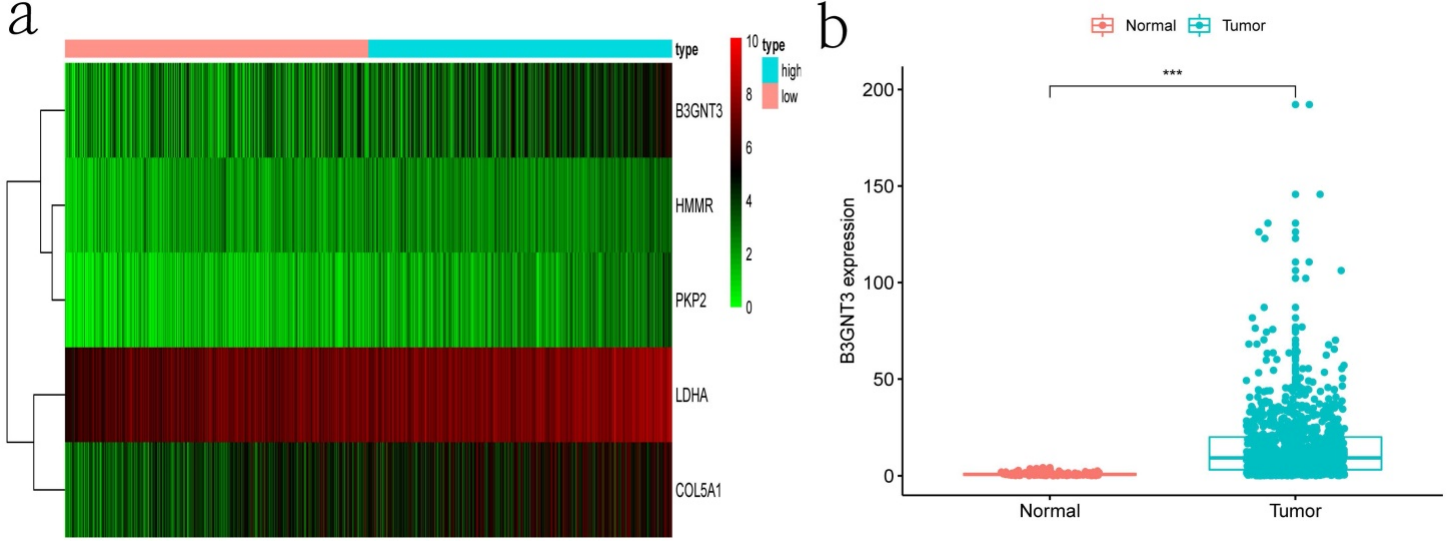

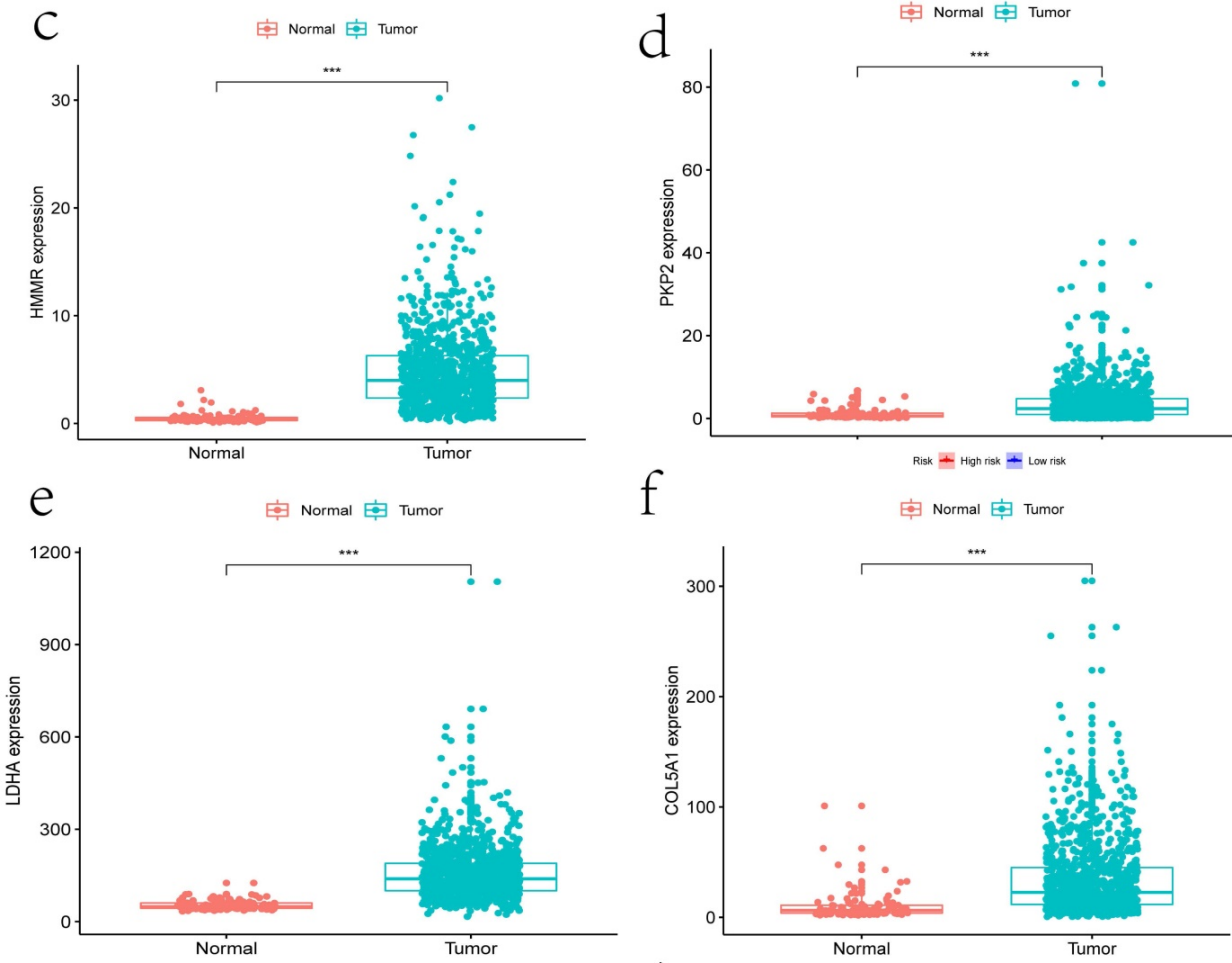

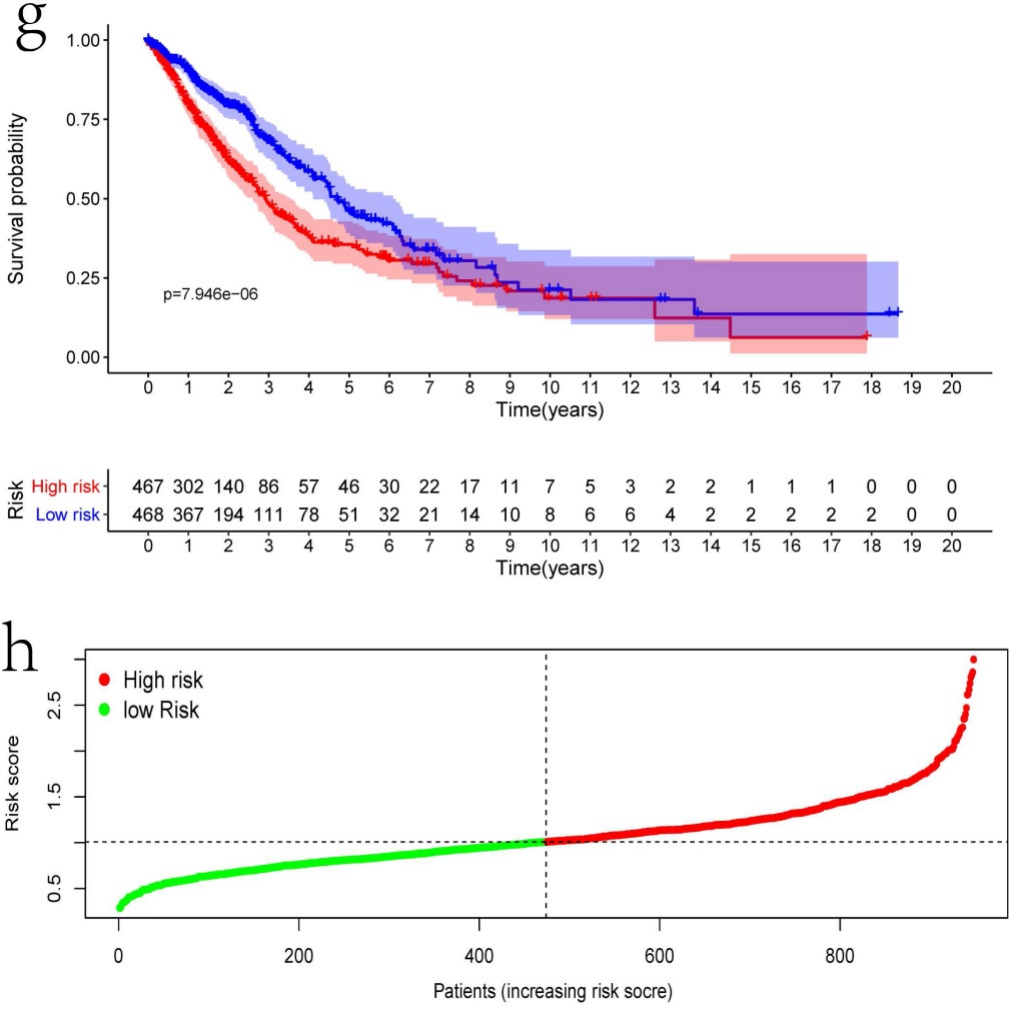

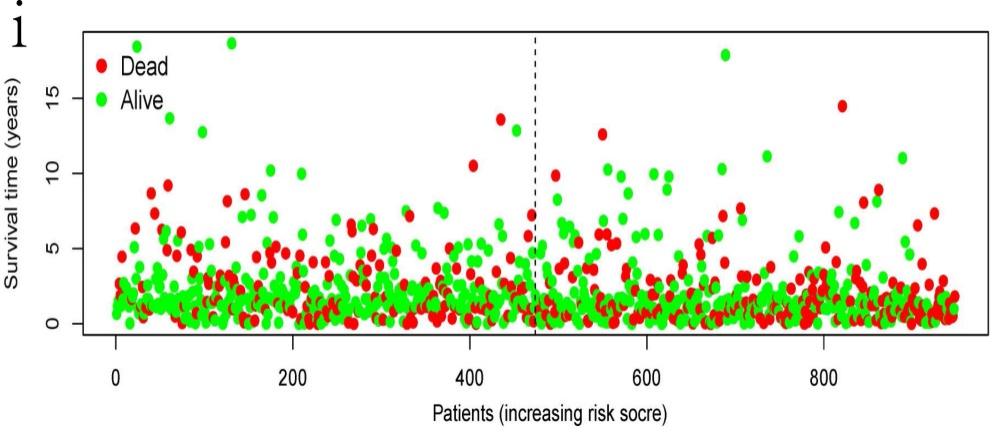
**The expression patterns of five genes in the signature. a)** Heatmap of five genes expression profiles. **b-f)** Different expression of five genes in the normal tissue and tumor tissue based on TCGA (*** represents *P*<0.001). **g)** Kaplan-Meier curve for NSCLC patients with high/low risk. **h)** Risk score distribution.** I)** Survival status distribution.

**Figure 4:**
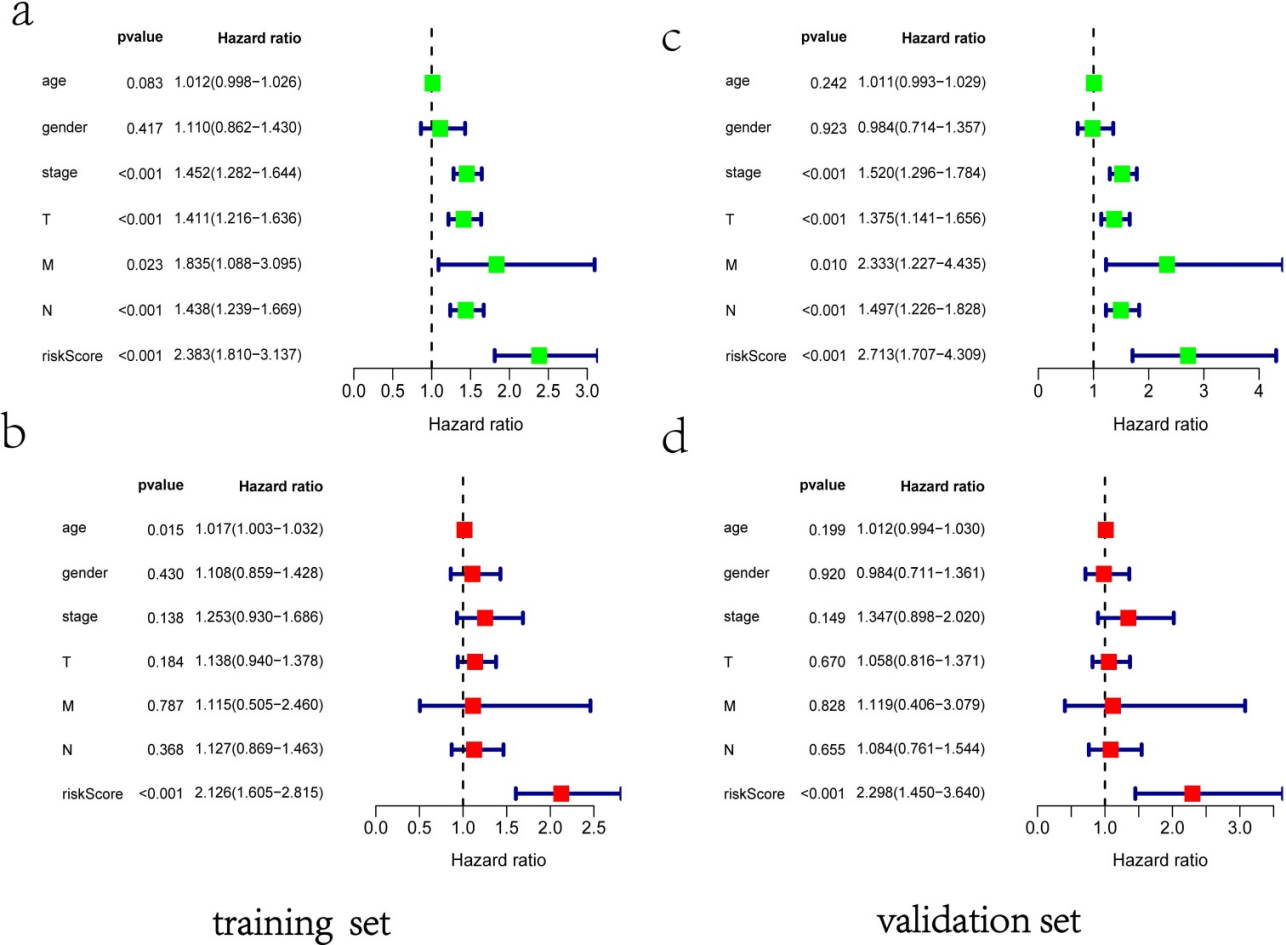
The independent prognostic significance of the signature was demonstrated by univariate and multivariate regression analysis.

Clinical information and corresponding gene expression data from 935 NSCLC patients were obtained from TCGA. GSEA results showed gene sets which were significantly enriched, with normalized p-values <0.05 and NES ≥1, including glycolysis, oxidative phosphorylation, DNA repair, inflammatory response, mitotic spindle, bile acid metabolism, E2F targets and G2M checkpoint (Table 2, Figure 1). We selected GLYCOLYSIS (P = 0.000, NES =2.46) for further analysis. Total 200 glycolysis related genes were obtained from GSEA, of which 46 genes were considered statistically significant (FDR < 0.05, |log2FC)| ≥1.5). The heatmap (Figure 2a) and boxplot **(**Figure 2b) showed the expression of these genes. A total of 10 genes expression pattern had a significant association with OS (P < 0.05) using univariate COX regression analysis. Subsequently, 5 of 10 genes were identified as independent prognosis genes to establish model using multivariate COX regression analysis. Finally, five prognosis genes and the expression coefficient of each gene were obtained, and a gene-based prognostic signature was constructed for calculating risk score of each NSCLC patient. The five genes were LDHA, HMMR, B3GNT3, PKP2, and COL5A1 (Table 3). The risk score = (0.2170 × Expression of LDHA) + (0.1200 × expression of HMMR) + (0.1088 × expression of B3GNT3) + (0.0960 × expression of PKP2) + (0.1344 × expression of COL5A1). The heatmap displayed the expression of five signature genes (Figure 3a). The five genes were all significantly up-regulated (Figure 3b-3f). Based on the median risk score, the NSCLC patients were divided into high- and low-risk groups. Patients of high-risk group tended to have a shorter survival time than those in low-risk group (P=7.946e-06) (Figure 3g). The risk score and survival state distribution were also visualized (Figure 3h, 3i). Furthermore, we used cox regression analysis to identify the independence of the risk score. Univariate independent prognostic analysis illustrated that TNM stage, T stage, N stage and risk score have statistical significance with OS (*P*<0.001) (Figure 4a). Multivariate independent prognostic analysis illustrated that risk score was an independent prognostic factor (*P*<0.001) (Figure 4b). Thus, our results confirmed that the glycolysis-related gene signature could be used as an independent prognostic factor in clinical practice (Table 4).

### Clinical correlation analysis

**Figure 5:**
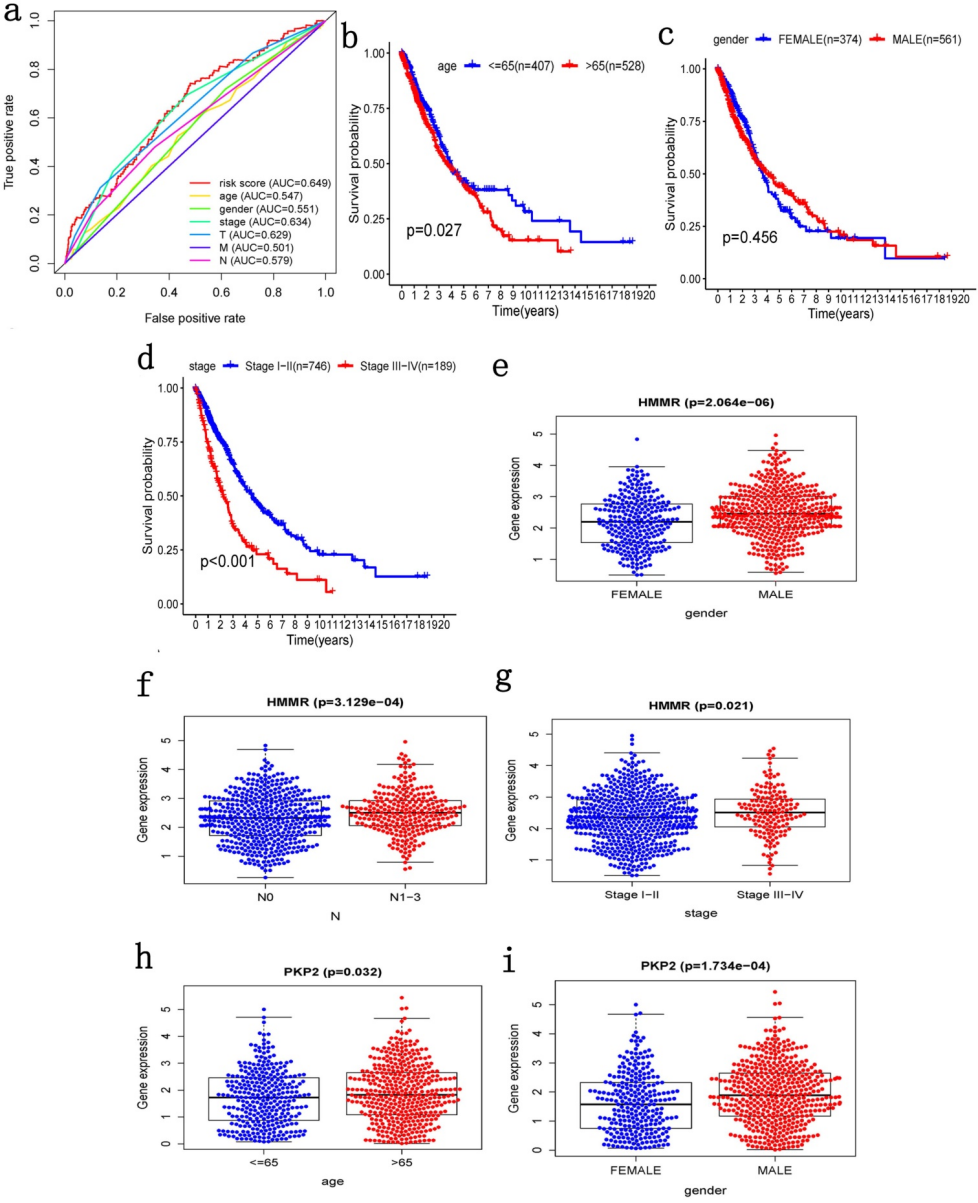

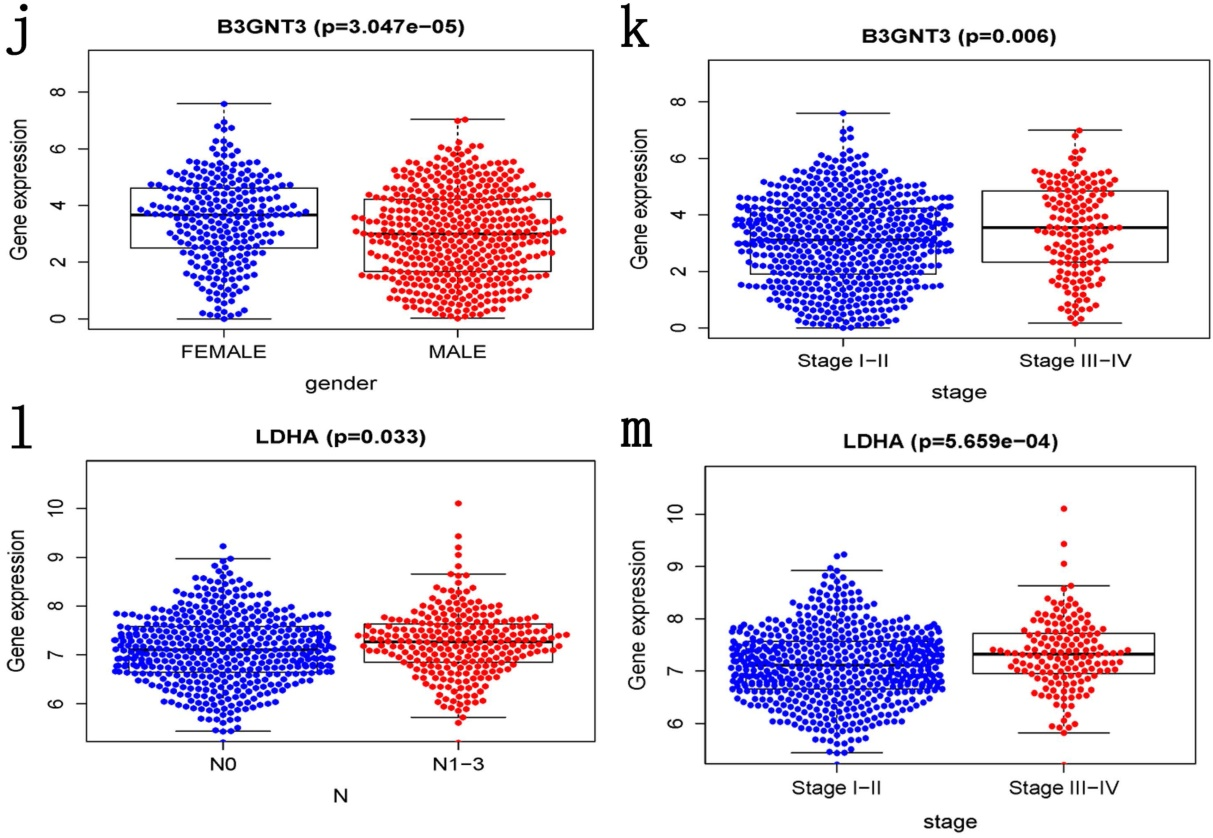
** Clinical correlation analysis. a)** The ROC analysis of OS for the signature and various clinical parameters**. b-d)** The statistical connection between signature and different clinical pathological factors. **e-m)** Five genes in signature with different expression among some clinical parameters.

ROC curves of OS helped to assess the prognosis significance of gene signature with AUC of 0.649, whereas lower scores were shown in other clinical parameters, such as age (AUC = 0.547), gender (AUC = 0.551), TNM stage (AUC = 0.634), T stage (AUC = 0.629), N stage (AUC = 0.579) and M stage (AUC = 0.501) (Figure 5a). The connection between signature and other clinical pathological factors was also used to demonstrate prognostic performance. Patients grouped by age (≤65, >65) or gender (male, female) or TNM stage (I-II, III-IV). Risk score was found to be significantly associated with age (p = 0.027) and TNM stage (*p* <0.001), but not with gender (Figure 5b-d). Additionally, these five genes in signature with showed substantial difference among some clinical parameters. Differential expression of HMMR showed across different gender, N stage and TNM stage (Figure 5e-g). The differential expression of PKP2 exhibited across age and gender (Figure 5h, 5i). B3GNT3 was expressed differently across different gender and TNM stage (Figure 5j, 5k). The differential expression of LDHA was found in N stage and TNM stage (Figure 5l, 5m).

### Stratification analysis

**Figure 6:**
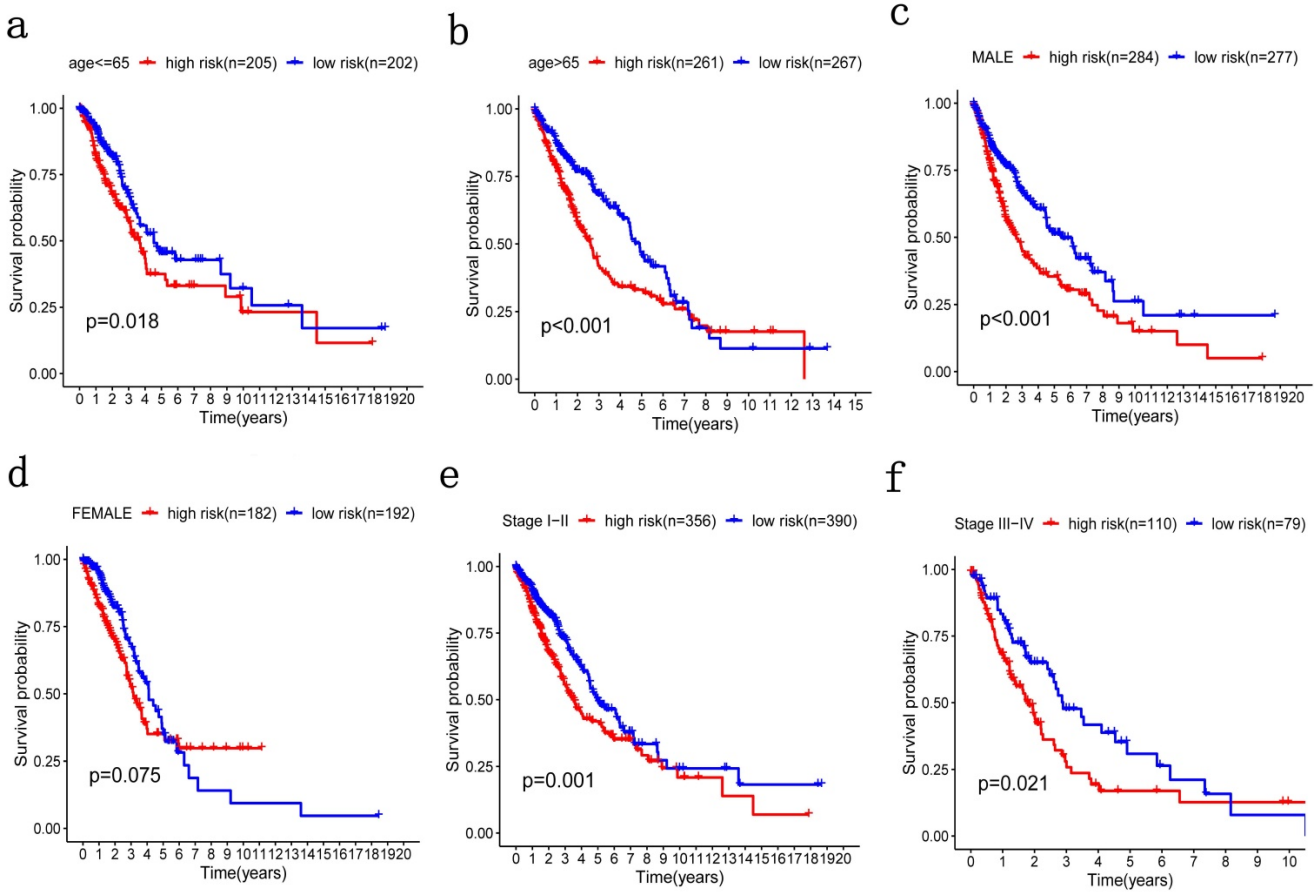
** Patients were grouped by different pathological parameters for stratification analysis. a-b)** Age; **c-d)** gender; and, **e-f)** stage.

Patients were grouped in the same manner as the previous step for stratification analysis where high-risk patients with poorer OS were grouped by age, no matter if they were older or younger than 65 (Figure 6a, 6b). The same was true of TNM stage (Figure 6e, 6f). Therefore, the potential associations between risk score with patients' age and TNM stage were statistically significant. However, there was no significant difference in gender stratification (Figure 6c, 6d).

### Prognostic significance in validation set

**Figure 7:**
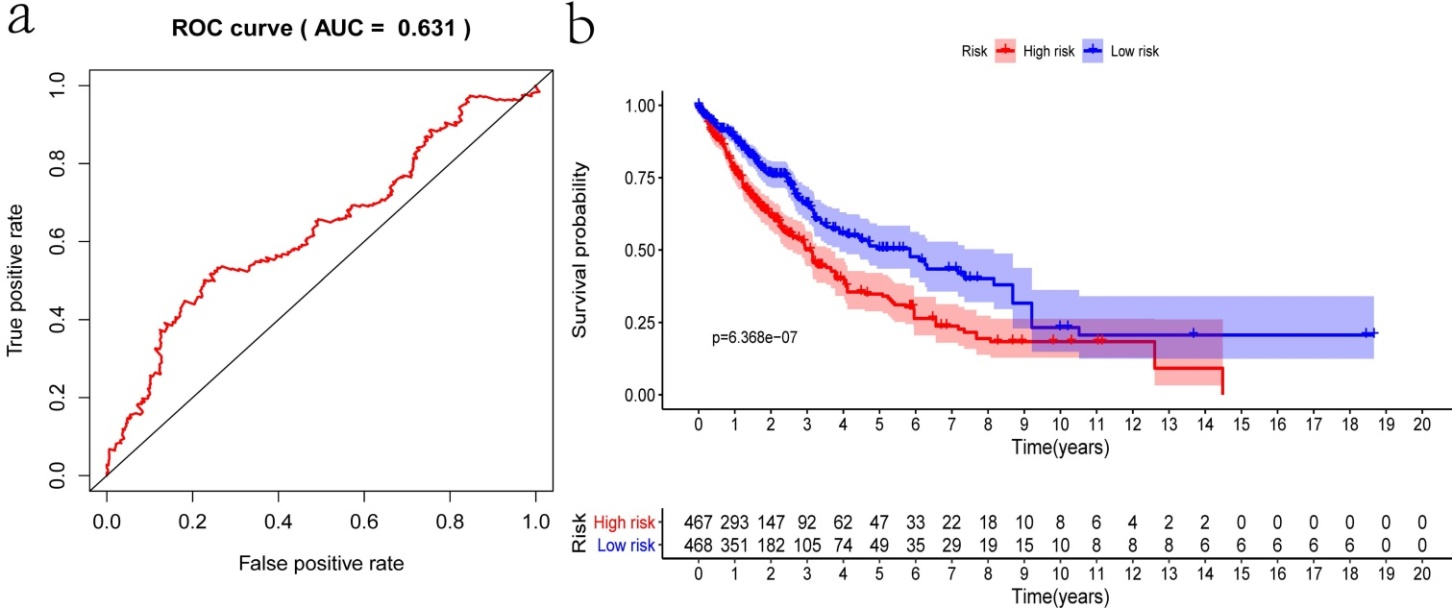

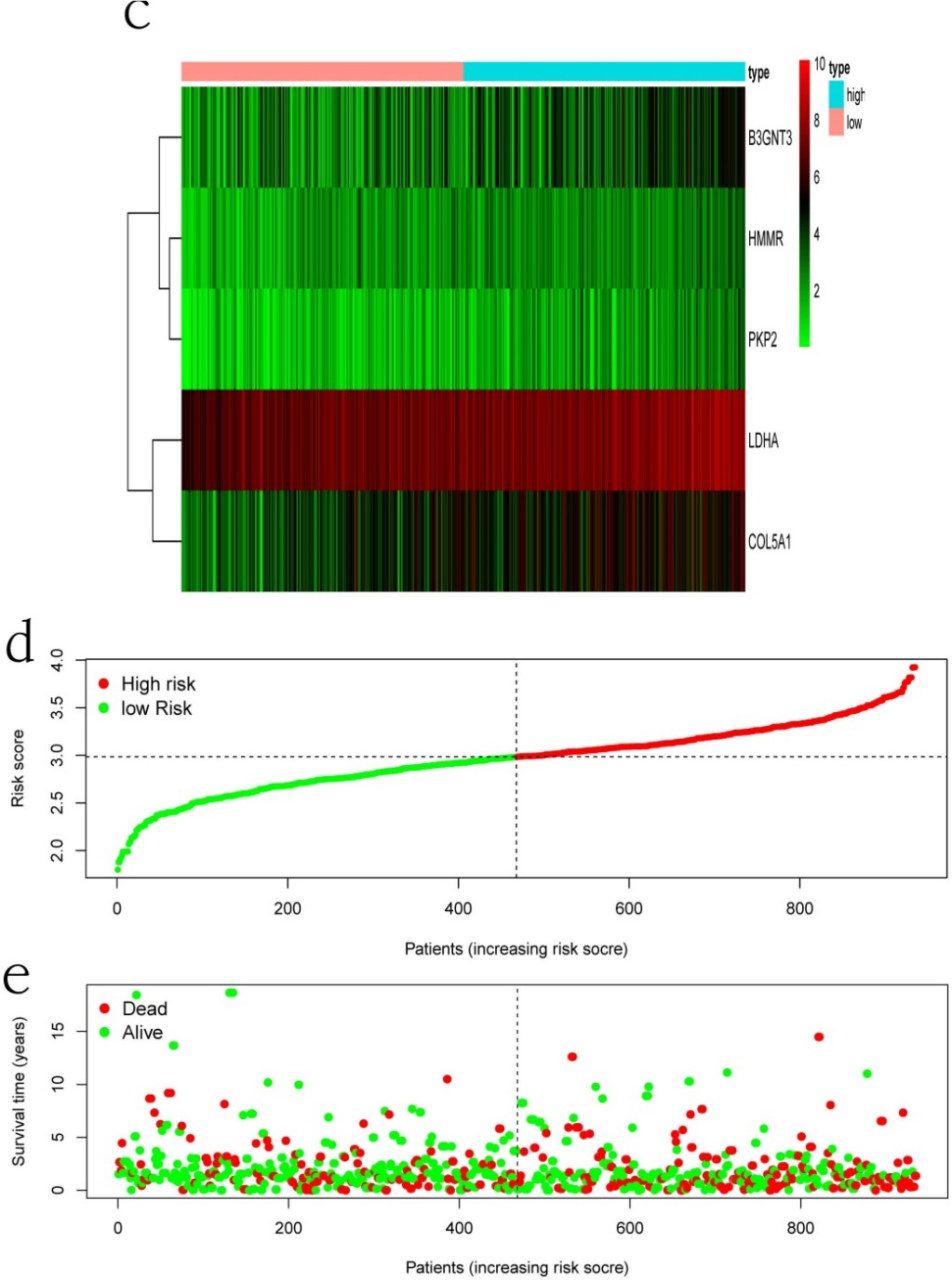
** Verification of the prognostic signature with the validation set. a)** ROC curve; **b)** Kaplan-Meier curve; **c)** Heatmap of five genes expression profiles in validation set; **d)** Risk score distribution;** e)** Survival status distribution.

Internal validation was conducted using the bootstrapping method. The original dataset was acted as training set. The validation set was performed as described above, including survival analysis, risk analysis, independent prognosis analysis and ROC curve drawing. The AUC of ROC was 0.631, which showed that this model has superior accuracy (Figure 7a). The OS was notably shorter in high-risk group than in the low-risk group (*P*-value=6.368e-07; Figure 7b). Besides, univariate and multivariate regression analysis were performed on the validation set to demonstrate the independent prognostic significance of the signature (Figure 4c, 4d). The heatmap, risk score curve and survival status data in validation set were shown in Figure 7c-e. The prognostic significance of our signature was further verified in the validation set.

### External validation using online database

**Figure 8:**
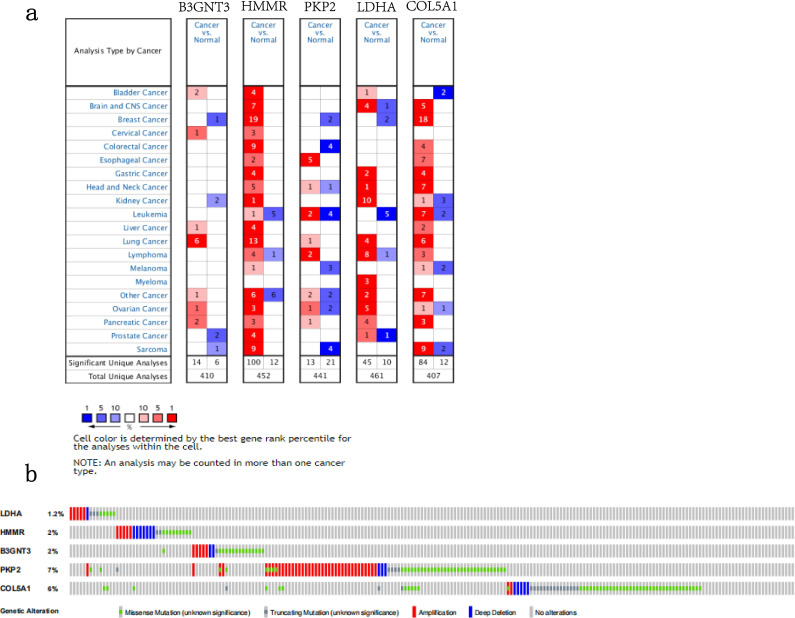
** The expression and alterations of the five prognostic genes. a.** The expression profiles of the five genes in the Oncomine database. **b.** The alteration proportion for the five genes in 1144 clinical samples of non-small cell lung cancer in the cBioPortal database.

**Figure 9:**
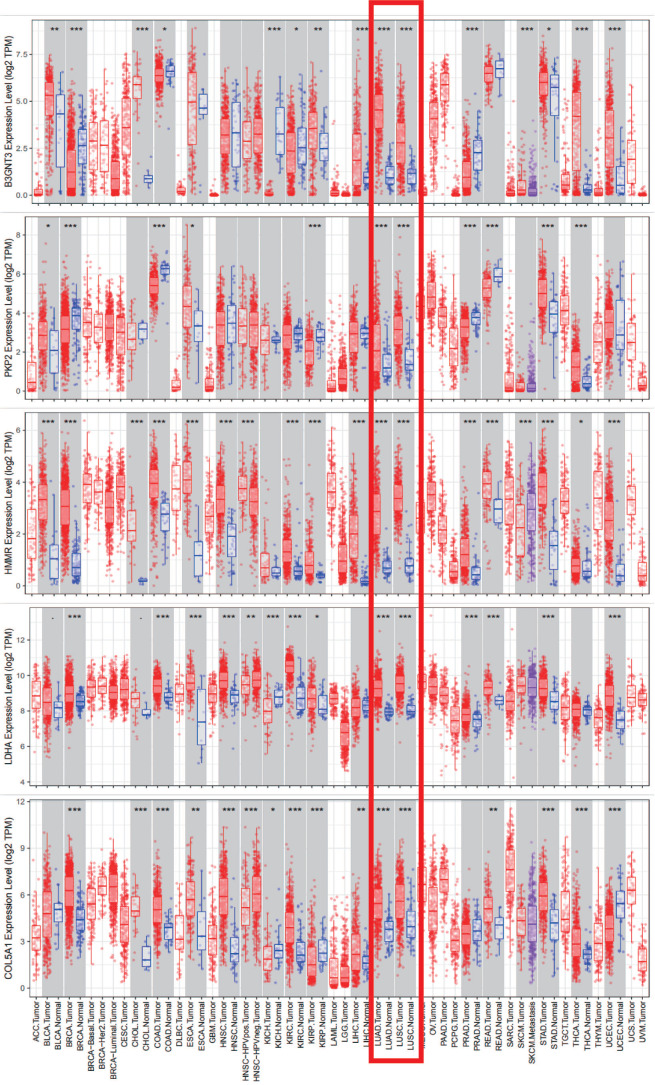
Five signature genes expression data from TIMER database.

We analyzed the alterations of the five genes in 1144 NSCLC samples in the cBioPortal for Cancer Genomics. The results showed that the genes in signature were mutated in 191 (17%) of the sequenced cases. As shown in Figure 8b.The five genes in the signature were all up-regulated in NSCLC.This is also confirmed in the Oncomine database (Figure 8a) and the Timer database (Figure 9).

## Discussion

NSCLC remains a major challenge for global public health, with incidence and mortality still increasing in many countries. Although great progress has been made, the underlying molecular pathogenesis of NSCLC is still unclear. Considering the poor prognosis of NSCLC due to its later diagnosis, identification reliable prognostic markers and establishment of more accurate prognostic models are urgently needed.

In recent years, the glycolysis mechanism in various diseases has caused wide attention 30-32. Growing evidence show that glycolysis key genes play an important role in regulating cancer cell metabolism and may be a potential therapeutic option 33,34. In lung cancer, glucose metabolism feature has also been further elucidated 35-37. For instance, significantly upregulated OTUB2 in NSCLC stimulated the Warburg effect and was closely related to metastasis, advanced tumor stages, poor survival, and recurrence 38. Transcription factor BACH1 stimulates glycolysis-dependent lung cancer metastasis by increasing glucose uptake, glycolysis rates, and lactate secretion 39.

In this study, we established a new predictive signature including five glycolysis related genes. Univariate and multivariate cox regression analysis determined the independent prognostic effect for predicting the risk of NSCLC. ROC curve and stratification analysis also proved the prognostic significance. More importantly, we established a validation set to verify the reliability of this signature. Similar studies have been published before. Zhang et al. and Liu et al. respectively identified glycolysis-related gene signature for predicting survival of lung adenocarcinoma patients 40,41. However, their signatures were not validated internally, which is the advantage of this study.

For the five signature genes, there have been some studies on its role in cancer. LDHA, a key enzyme which catalyzes the formation of lactic acid from pyruvic acid, is a key gene in aerobic glycolysis and is widely seen as a therapeutic target for cancer 42. Phosphorylated LDHA could promote head and neck cancer and breast cancer cells invasion and metastasis 43. Besides, LDHA inhibitors can be used together with other chemotherapy drugs to play a synergistic role in anti-tumor 44. HMMR plays an important role in neural development by correcting spindle position. HMMR knockout mice suffer defective neural development 45. Furthermore, the overexpression of HMMR in numerous tumor types is also associated with tumor relapse and propagation. HMMR also supports the cancer stem cell properties in gastric cancer and glioblastoma 46,47. HMMR might be a potential prognostic marker and therapeutic target 48,49. Some studies have suggested that the B3GNT3 were significantly overexpressed in various tumors 50,51. The expression level of B3GNT3 in patients with NSCLC or early-stage cervical cancer was associated with unfavourable clinicopathological parameters. These patients with high B3GNT3 expression had a shorter OS and disease-free survival (DFS) compared with those with low expression 52,53. In contrast, B3GNT3 predicts a favorable cancer behavior of neuroblastoma 54. In addition, down-regulation of B3GNT3 can enhance cytotoxic T-cell-mediated anti-tumor immunity in triple-negative breast cancer 55. COL5A1 is collagen family member that encodes an alpha chain for one of the low abundance fibrillar collagens. Upregulated COL5A1 indicated poor prognosis in breast cancer, clear cell renal cell carcinoma, lung adenocarcinoma and tongue squamous cell carcinoma 56-59. PKP2, a member of the arm-repeat protein family, is important for the assembly of junctional proteins. But as far as we know, the mechanism of PKP2 in cancer is still unclear.

The risk score was based on mRNA expression which could be easily acquired. The signature can effectively evaluate the prognosis of NSCLC patients and may be a complement to CT and pathological methods. Despite practical independent prognosis of our results, our study leaves much to be desired. First, our study only involved 200 glycolysis related genes, not the entire mRNA expression profiles. Second, validating the signature in a larger independent external set is necessary. Third, cell and animal experiments are needed to clarify specific mechanism of the five mRNAs in the regulation of tumor glycolysis. Nevertheless, the present study provides a novel suggestion for predicting prognosis of NSCLC patients from the perspective of glycolysis.

## Conclusion

In summary, we recognized five glycolysis related genes associated with OS of NSCLC patients, and constructed a risk score signature to make a survival prediction. Our risk score model could distinguish NSCLC patients with different survival outcomes, which may contribute to the clinical decision of individualized treatment program.

## Figures and Tables

**Table 1:** Clinical characteristics of included patients with non-small cell lung cancer (NSCLC)

Clinical characteristic	N	Percentage (%)
**Age (years)**		
≤65	407	43.5
>65	528	56.5
**Gender**		
Male	561	60.0
Female	374	40.0
**TNM stage**		
I-II	746	79.8
III-IV	189	20.2
**T stage**		
T1-2	785	84.0
T3-4	150	16.0
**N stage**		
N0	599	64.1
N1-3	336	35.9
**M stage**		
M0	697	74.5
M1-3	238	25.5

**Table 2:** Gene sets enriched in non-small cell lung cancer (NSCLC)

GS follow link to MSigDB	SIZE	NES	NOM *p*-value	Rank at MAX
Glycolysis	200	2.46	0	9022
Oxidative phosphorylation	200	1.84	0.034	8207
DNA repair	150	2.16	0	8719
Inflammatory response	200	-1.88	0.024	6162
Mitotic spindle	198	1.81	0.014	5517
Bile acid metabolism	112	-1.68	0.015	6354
E2F targets	198	2.18	0	4960
G2M checkpoint	196	2.18	0	7277

**Table 3:** The information of five mRNAs involved in the signature

mRNA	Ensemble ID (ENSG0000)	Location	coef	HR	HR.95L	HR.95H	*p*-value
LDHA	0134333	Chr11: 18, 394, 389-18, 408, 425	0.21695	1.24228	1.05311	1.46544	0.01006
B3GNT3	0179913	Chr19: 17, 794, 828-17, 813, 576	0.10884	1.11498	1.03914	1.19636	0.00246
PKP2	0057294	Chr12: 32, 790, 745-32, 896, 846	0.09602	1.10067	0.98912	1.22481	0.07853
COL5A1	0130635	Chr9: 134, 641, 774-134, 844, 843	0.13439	1.14384	1.05674	1.23811	0.00088
HMMR	0072571	Chr5: 163, 460, 203-163, 491, 945	0.12004	1.12754	0.96947	1.31138	0.11932

**Table 4:** Univariate and multivariate analysis for each clinical feature

Clinical feature	Univariate analysis		*P*	Multivariate analysis		*P*
	HR	95%CI		HR	95%CI	
Age	1.012	0.998-1.026	0.083	1.017	1.003-1.032	0.015
Gender	1.11	0.862-1.43	0.417	1.108	0.859-1.428	0.430
Stage	1.452	1.282-1.644	<0.001	1.253	0.93-1.686	0.138
T	1.411	1.216-1.636	<0.001	1.138	0.94-1.378	0.184
M	1.835	1.088-3.095	0.023	1.115	0.505-2.46	0.787
N	1.438	1.239-1.669	<0.001	1.127	0.869-1.463	0.368
Risk score	2.383	1.810-3.137	<0.001	2.126	1.605-2.815	<0.001

## Abbreviations

NSCLC: Non-small cell lung cancer
TCGA: The Cancer Genome Atlas
GSEA: Gene Set Enrichment Analysis
OS: Overall survival
ROC: Receiver operating characteristic
NES: Normalized enrich score
FDR: False discovery rate
TIMER: Tumor Immune Estimation Resource
DFS: Disease-free survival
